# Mid-Term outcomes following fresh-frozen humeral head osteochondral allograft reconstruction for reverse Hill Sachs lesion: a case series

**DOI:** 10.1186/s12891-021-04657-z

**Published:** 2021-09-08

**Authors:** Giulio Maria Marcheggiani Muccioli, Vito Gaetano Rinaldi, Giada Lullini, Alice Ritali, Massimiliano Mosca, Matteo Romagnoli, Enrico Guerra, Stefano Zaffagnini

**Affiliations:** 1 II Clinica Ortopedica e Traumatologica, IRCCS Istituto Ortopedico Rizzoli, Bologna, Italy; 2 DIBINEM, University of Bologna, via Pupilli, 1 c/o IRCCS Istituto Ortopedico Rizzoli, 40136 Bologna, Italy; 3 UOC Medicina Riabilitativa e Neuroriabilitazione, IRCCS Istituto delle Scienze Neurologiche, Bologna, Italy; 4 Chirurgia della Spalla e del Gomito, IRCCS Istituto Ortopedico Rizzoli, Bologna, Italy

**Keywords:** Segmental reconstruction, Humeral head, Locked posterior dislocation, Long-term, Humeral head allograft, fresh-frozen

## Abstract

**Background:**

Locked posterior glenohumeral dislocations with a reverse Hill-Sachs impaction fracture involving less than 30% of the humeral head are most frequently treated with lesser tuberosity transfer into the defect, whereas those involving more than 50% undergo humeral head arthroplasty. Reconstruction of the defect with segmental femoral osteochondral allografts has been proposed to treat patients between these two ranges, but the medium−/long-term outcomes of this joint-preserving procedure are controversial.

**Methods:**

Between 2001 and 2018, 12 consecutive patients with a unilateral locked posterior shoulder dislocation and an impaction fracture from 30 to 50% (mean 31% ± 1.32) of the humeral head were treated with segmental reconstruction of the defect with fresh-frozen humeral head osteochondral allografts. Patients were assessed clinically, radiographically and with computed tomography (CT) at a medium follow-up of 66 ± 50.25 months (range, 24–225).

**Results:**

All twelve shoulders presented a slight limitation in anterior elevation (average, 166.6° ± 22.76). The mean active external rotation with the shoulder at 90° of abduction was 82.5° ± 6.61, and that with the arm held in stable adduction was 79.16 ± 18.80. The mean abduction was 156.25° ± 25.09.

The mean Constant-Murley score (CS) was 82 ± 15.09 points (range, 40–97 points), and the mean ASES was 94 ± 8.49 points. The mean pre- and postoperatively Western Ontario Shoulder Instability index (WOSI) was 236.5 ± 227.9 and 11.20 ± 10.85, respectively.

Development of osteoarthrosis (OA) was minimal. The average allograft resorption rate was 4% ± 2.4. There were no cases of failure (reoperation for any reason) in this series.

**Conclusion:**

Segmental humeral head reconstruction with humeral head fresh-frozen osteochondral allografts provides good to excellent clinical results with low-grade OA and low allograft resorption in patients with locked posterior shoulder dislocation.

**Trial registration:**

ClinicalTrials.gov PRS, ClinicalTrials.gov ID: NCT04823455. Registered 29 March 2021 - Retrospectively registered, https://register.clinicaltrials.gov/prs/app/action/SelectProtocol?sid=S000AU8P&selectaction=Edit&uid=U0004J36&ts=12&cx=6cykp8

**Level of evidence:**

Level IV, Case Series, Treatment Study.

## Background

Posterior dislocation of the shoulder is a rare injury and is usually associated with a reverse Hill-Sachs lesion, which is also known as a McLaughlin impression fracture of the humeral head [1].

Posterior dislocations can often remain unrecognized and become chronic because of the similarities of the lesion with that of a common condition called frozen shoulder [2].

Chronic dislocations, defined as those with a diagnostic delay of at least six weeks, are associated with osteopenia of the humeral head due to loss of contact with the glenoid, leading to degenerative changes of the irregular glenohumeral joint. In up to 79% of cases, the diagnosis is made only once the injury has become chronic and the shoulder has been locked, negatively affecting the prognosis [3].

Because of the limited number of patients with locked posterior shoulder dislocations, to the best of the authors’ knowledge, no large patient cohort studies or evidence-based treatment strategies have been reported in the literature.

Gerber et [4] al reported the largest cohort study with the longest follow-up on this topic. They employed femoral head osteochondral allografts to reconstruct large defects (affecting from 30 to 40% of the humeral head articular surface), reporting mild or severe osteoarthritis (OA) in approximately half of the cases at the 5-year follow-up.

The aim of the present study was to evaluate a series of consecutive patients with reverse Hill-Sachs lesions after an acute locked posterior glenohumeral dislocation treated with segmental reconstruction using fresh-frozen humeral head osteochondral allografts.

The hypothesis of the present study is that the spherical shape of the humeral head will be restored by fresh-frozen humeral head osteochondral allograft reconstruction with consequent low-grade bone reabsorption and OA at a medium follow-up of 66 months.

## Methods

Between 2001 and 2018, 12 consecutive patients suffering from a locked posterior glenohumeral dislocation with an impaction fracture from 30 to 50% (mean 31% ± 1.32) of the humeral head diameter were surgically treated. During surgery, the bone defect was substituted with a fresh-frozen humeral head osteochondral allograft. All twelve shoulders were treated acutely (according to a review of the literature, a dislocation was considered to be acute when the duration was less than three weeks [5]). The patients were retrospectively reviewed clinically and radiographically at a medium follow-up of 66 ± 50.25 months (range, 24–225).

The mean patient age at the time of shoulder dislocation was 54.8 ± 12.4 years (range, 31–72 years).

The inclusion criterion was a diagnosis of an acute nonreducible posterior glenohumeral dislocation with an associated McLaughlin lesion affecting more than 30% of the cartilaginous circumference of the humeral head.

Patients with associated injuries to the affected upper limb, with neuromuscular or psychomotor disorders or with disorders affecting connective tissues were excluded from the study.

### Radiographic measurement

**Fig. 1:**
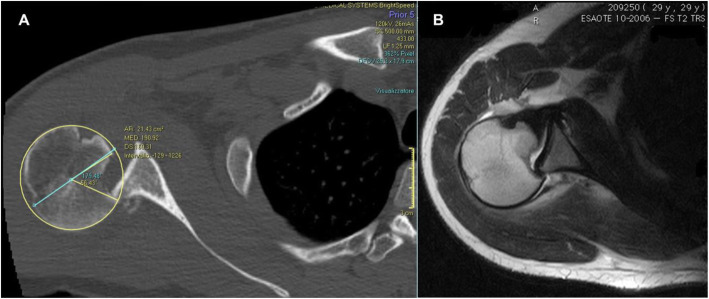
The defect angle (DA) and the Cartilage Angle (CA) are calculated on a CT slice taken at or immediately below the coracoid tip. The percentage of the CA (179° in this case) that the DA (56° in this case) represents is the estimated size of the defect (31% in this case)

Once conventional radiography confirmed the diagnosis, Computed tomography (CT) and magnetic resonance imaging (MRI) have been employed for pre-operative investigation. On CT scan, a head fracture involving at least 30% of the cartilage circumference without rotator cuff tendon avulsion tears or significant posterior glenoid rim fractures was reported. The extension of humeral head defect was measured on the preoperative CT scan by defining the cartilage angle (CA) and the defect angle (DA) according to Gerber et al. [4]. On the CT scan, a circle was placed over the humeral head at -or immediately- below the level of the coracoid. The CA was measured by drawing a line, the latter beginning from the centre of the circle to the cartilage neighboring the lesser tuberosity, apart from an additional line from the centre of the circle to the posterior end of the cartilage, this one being nearby the infraspinatus insertion. The DA was determined as the angle resulting from the lines linking the defect’s anterior, posterior limit and the centre of the humeral head. The assessed magnitude of the humeral head defect was the cartilage angle percentage that the defect angle embodied (Fig. 1). A posterior glenoid rim defect greater than half of the largest anteroposterior diameter was considered to be relevant [6].

### Surgical technique

**Fig. 2:**
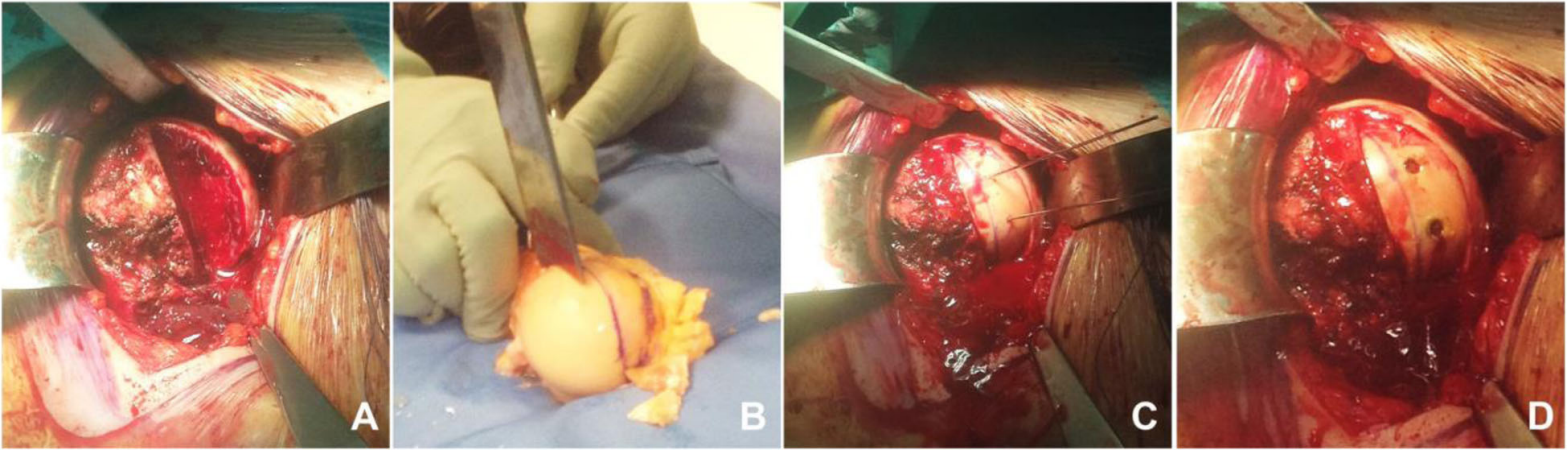
Reverse Hill-Sachs lesion reconstruction with segmental humeral head osteochondral allograft operative Technique. Humeral head defect (**A**), Shaping of humeral head osteochondral allograft (**B**),defect restored and fixed with two titanium cancellous bone lag screw (**C,D**)

Patients were placed in the beach-chair position and operated on under general anesthesia for the best relaxation. The glenohumeral joint was accessed using a deltopectoral approach. When possible, the cephalic vein was conserved and laterally shifted. The subscapularis tendon was completely detached approximately 1 cm from its insertion on the lesser tuberosity. The tendon extremity was marked with two no. 2 Ethibond sutures (Ethicon, Somerville, NJ, USA). The axillary nerve and the anterior circumflex vessels were carefully protected. To ensure accurate exposure of the humeral head, a capsulotomy was performed, and the superior glenohumeral ligament and the coracohumeral ligament were divided. Once the capsule was released, reduction was gained by putting internally rotating the arm and pulling the head sideways with a lever inserted in the anteromedial defect of the humeral head. After reduction, the humerus was stabilized in neutral rotation. Intraoperatively, the internal rotation test led to immediate posterior redislocation, making segmental humeral head reconstruction necessary for each patient. A half-moon shaped, segmental fresh-frozen humeral head osteochondral allograft from XXXXX Institute’s Cell and Musculoskeletal Tissue Bank was contoured to fit the segmental defect and restore the original shape of the humeral head. The graft was fixed with two 3.5-mm titanium cancellous bone lag screws oriented toward the greater tuberosity (Fig. 2). The humeral head was reduced using a Cobb elevator with a soft maneuver, paying attention to avoid causing further damage to the humeral head and glenoid. This anatomical restoration of the humeral shape avoided intraoperative redislocation in all treated cases. The anterior capsule was repaired with no. 2 Ethibond absorbable sutures (Ethicon, Somerville, NJ, USA). The subscapularis was repaired using 2 Corkscrew titanium anchors with 4 mattress stitches. The rotator interval was not closed. Superficial layers were closed after placing one suction drain.

### Postoperative rehabilitation

After surgery, the arm was positioned in a brace in neutral rotation and at 15 degrees of abduction. A minimum of 4 weeks of activity restriction was prescribed to minimize stress on healing structures. During this period of limited upper extremity use, we recommended active exercise for the noninvolved joints (elbow, wrist, and hand) followed by a 60-day standard rehabilitation program. Sports activity was allowed after 4 months.

### Outcome measures

**Fig. 3:**
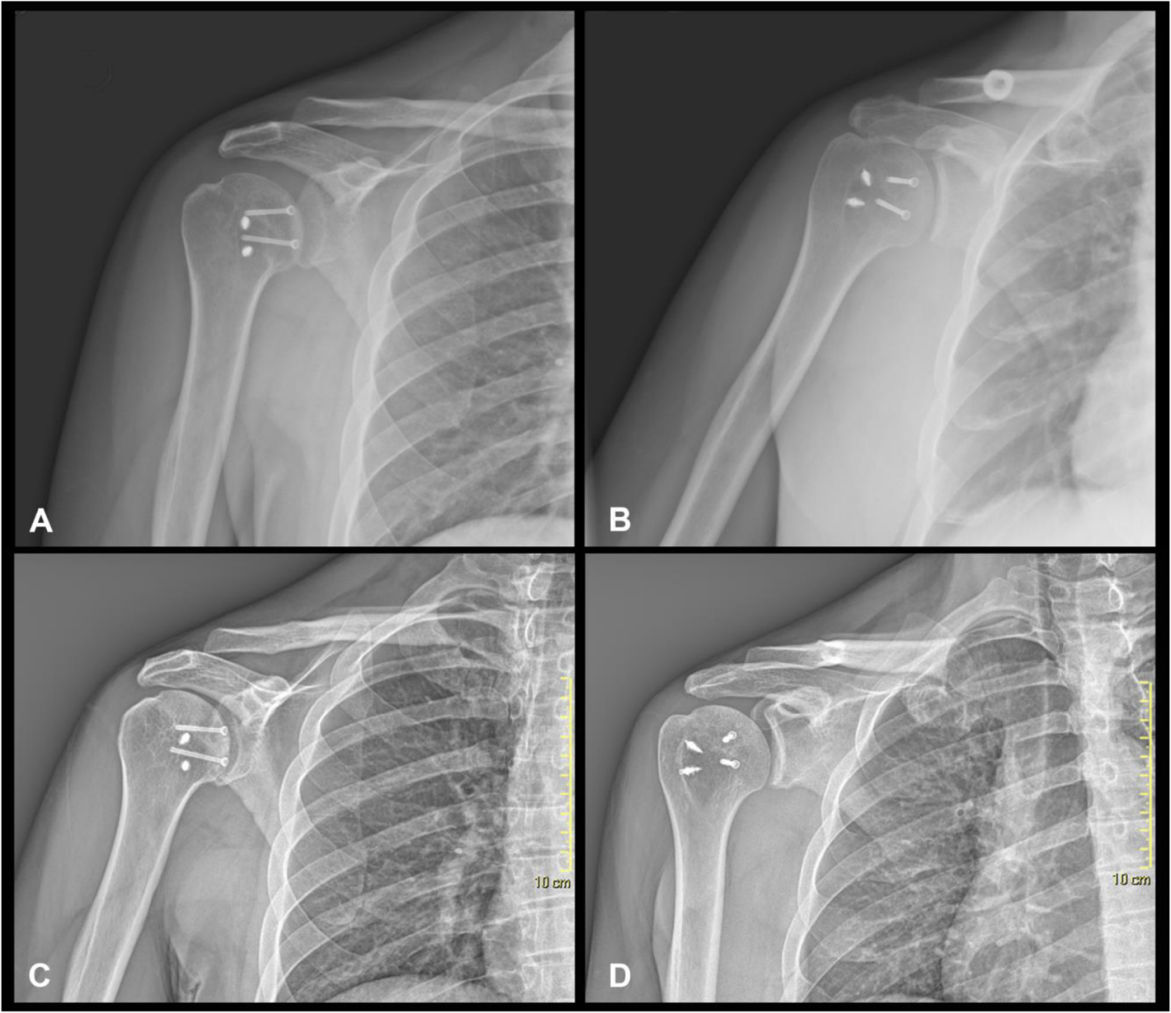
Standard shoulder Anteroposterior X-Ray of the same patients taken post-operative (**A,B**) and at 65 months follow-up (**C,D**). Osteoarthritis (OA) was graded according to Samilson-Prieto Score (Grade I in this case)

**Fig. 4:**
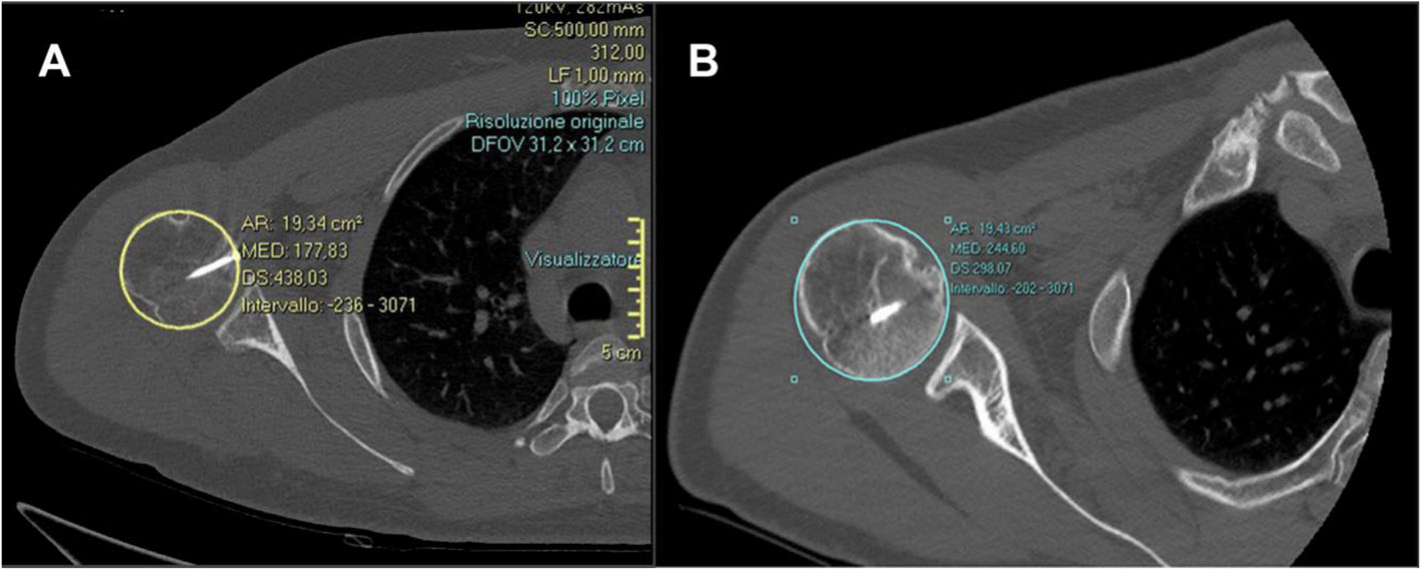
Allograft resorption rate calculation adapting defect determination technique proposed by Gerber et al. for humeral head defect calculation by defining the CA and the DA. Allograft resorption is 0% in this case

Included patients were clinically and radiographically re-evaluated for the purpose of this study by examiners not involved in the primary treatment at a mean of 66 ± 50.25 months (range, 24–225 months) postoperatively. The clinical examination consisted of a physical examination and structured interview. The range of motion of the affected shoulder was recorded, including anterior elevation, abduction, medial rotation, and external rotation with the arm at 90 degrees of abduction. Reliable and validated scoring systems were administered, including the Western Ontario Shoulder Instability index (WOSI) [7], the Constant-Murley score (CS) [8, 9], and the American Shoulder and Elbow Surgeons Shoulder Score (ASES) [10]. Computed tomography was carried out at the medium follow-up of 66 months in all patients to evaluate OA progression and allograft resorption. OA was graded at the same follow-up on standard X-rays as mild (grade I), moderate (grade II) and severe (grade III) according to the Samilson-Prieto score [11] (Fig. 3). The allograft resorption rate was calculated for all twelve shoulders by adapting the calculation technique proposed by Gerber et al. for humeral head defect determination by defining the CA and the DA [6] (Fig. 4).

Reoperation for any reason (revision, conversion to arthroplasty, etc.) was considered as treatment failure.

The present study has been promoted by Istituto Ortopedico Rizzoli, Bologna (Italy) and approved by the Local Ethics Committee of “Area Vasta Emilia Centro” (reference number: 0007888).

### Statistical analysis

The collected data were analyzed using SPSS. Continuous variables are expressed as the mean ± standard deviation, while categorical variables are expressed as percentages with 95% confidence intervals. To test the normality of the sample, the chi-square test was used for categorical variables, and the Kolmogorov-Smirnov test was used for continuous variables. Differences in variables of interest between pre- and postoperatively were analyzed with parametric (t-test) or nonparametric (Wilcoxon rank) tests for dependent samples. Univariate and multivariate analyses with possible linear and logistic regressions were conducted to test the correlations between baseline and outcome variables and to check possible confounding factors.

A *p*-value of less than 0.05 was considered significant for all analyses.

## Results

Preoperatively, all patients were locked in external rotation. This condition did not allow a clinical examination or the administration of clinical outcome scales.

At the follow-up physical examination, all patients presented a slight limitation in anterior elevation and decreased external rotation of the operated shoulder compared to the contralateral shoulder. The mean active elevation was 166.6° ± 22.76, the mean active external rotation with the shoulder abducted 90° was 82.5° ± 6.61, and that with the arm held in stable adduction was 79.16 ± 18.80. The mean active shoulder abduction was 156.25° ± 25.09. Range-of-motion results are reported in Table 1.

**Table 1:** Range of motion

PATIENT	ELEVATION(°)	ER1(°)	ER2(°)	ABD(°)
**1**	180	80	180	150
**2**	150	90	150	180
**3**	170	85	170	150
**4**	180	70	180	150
**5**	180	85	180	150
**6**	100	80	100	85
**7**	180	90	180	180
**8**	175	70	175	160
**9**	150	90	150	150
**10**	180	85	180	180
**11**	180	85	180	180
**12**	175	80	175	160
**MEAN VALUE ± SD**	166,6 ± 22.76	82,5 ± 6.61	79,16 ± 18.80	156,25 ± 25.09

Post-operative ROM at 66 months medium follow-up. ER1:active external rotation with the shoulder 90° abducted; ER2:active external rotation with the arm held in stable adduction. ABD:abduction

Clinical outcomes are reported in Table 2. The mean CS was 82 ± 15.09 points (range, 40–97 points), and the mean ASES was 94 ± 8.49 points. The mean pre- and postoperatively WOSI was 236.5 ± 227.9 and 11.20 ± 10.85, respectively.

**Table 2:** Clinical outcomes

PATIENT	COSTANT-MURLEY	WOSI	ASES
**1**	84	165 (7,85%)	95
**2**	97	103 (4,90%)	98
**3**	76	324 (15,40%)	83
**4**	87	270 (12,80%)	98
**5**	89	155 (7,38%)	98
**6**	40	963 (45,80%)	70
**7**	97	100 (4,80%)	100
**8**	89	156 (7,40%)	98
**9**	84	165 (7,85%)	95
**10**	97	103 (4,90%)	98
**11**	97	178 (7,95%)	100
**12**	89	156 (7,40%)	98
**MEAN VALUE± SD**	82,37± 15.09	236,5±227.9 (11,20%±10.85)	94,25± 8.49

Post-operative clinical scores at 5 years medium follow-up

According to the Samilson-Prieto score, the development of osteoarthrosis was minimal (10 patients grade 1; 2 patients grade 2). The average allograft resorption rate was 4% ± 2.4. Radiological results are reported in Table 3.

**Table 3:** Radiological scores

PATIENT	SAMILSON-PRIETO SCORE	REABSORPTION RATE
**1**	GRADE I	2%
**2**	GRADE I	2%
**3**	GRADE II	3%
**4**	GRADE I	10%
**5**	GRADE I	2%
**6**	GRADE II	10%
**7**	GRADE I	0%
**8**	GRADE I	2%
**9**	GRADE I	2%
**10**	GRADE I	3%
**11**	GRADE I	2%
**12**	GRADE I	2%
**MEAN VALUE ± SD**		4% ± 2.4

Post-operative radiological scores based on CT scan at 66 months medium follow-up

There were no cases of failure (reoperation for any reason) in this series.

## Discussion

The most important finding of the present study is that humeral head osteochondral allograft reconstruction for reverse Hill-Sachs lesions showed significant functional improvement with low-grade osteoarthritis (OA) and a low reabsorption rate at a mean of 66 ± months follow-up.

Posterior shoulder dislocation treatments available in the literature are mostly based on the percentage of humeral head bone loss. When this bone loss is less than 25%, posterior dislocations are mostly managed with closed reduction associated with posterior capsular arthroscopic repair with or without remplissage. Dislocations with humeral head bone loss ranging from 25 to 50% are mainly managed with open reconstruction with bone grafting or subscapularis tendon transfer (McLaughlin technique [2]). In cases of humeral head bone loss greater than 50%, arthroplasty is the first choice [12].

Few studies have investigated the outcome of segmental reconstruction of the humeral head using fresh-frozen osteochondral allografts.

To date, this is the first study in which humeral head defects resulting from a locked posterior dislocation were reconstructed using fresh-frozen humeral head osteochondral allografts. Previously, only Miyazaki et al. [13] proposed reconstruction of the humeral head with a humeral head allograft, although for humeral head osteonecrosis.

Gerber et al. [4] investigated twenty-one consecutive patients at a minimum of 10 years after humeral head reconstruction with femoral head osteochondral allografts or iliac crest autografts. They included patients with locked posterior dislocations with humeral head defects affecting at least 30% of the humeral head (mean, 43%; range, 30–55%). At the 10-year follow-up, treated shoulders showed good overall clinical results, with medium-high levels of osteoarthritis (OA) and a failure rate of 11% over the long term.

Diklic et al. [14] reviewed 13 consecutive segmental allograft reconstructions of the humeral head with defects affecting 25 to 50% of the humeral head at a mean follow-up of 4.5 years. They reported no recurrence of instability; osteonecrosis of the humeral head occurred in one patient, while the other 12 patients showed excellent results, for an overall CS of 87%.

In agreement with the present study, previously reported studies have shown improvements in clinical outcomes from pre- to postoperatively. However, they have also reported higher failure rates and higher degrees of arthrosis in the long term.

These contrasting results could be attributed to the different reconstruction technique employed in this study. Gerber et al. [4] and Diklic et al. [14] employed femoral head allografts or iliac crest autografts to treat bone defects.

In the present study, only humeral allograft heads were used for segmental reconstruction, and as reported in previous studies, the radius of curvature of this type of graft is very different from that of femoral head or iliac crest grafts. In the literature, the average radius of curvature in the frontal plane (ROCF) of the humeral head is 25.4 mm. The average radius of curvature in the sagittal plane (ROCS) is 23.8 mm [15] The mean femoral head radius is 22.0 ± 1.3 mm, differing from that of the humeral head [16].

Another possible explanation of these differences could be the fact that Gerber et al. [4] and Diklic et al. [14] treated patients with humeral defects affecting up to 55 and 60% of the humeral head, respectively. In the present study, only defects affecting up to 35% of the humeral head were treated with segmental reconstruction, while humeral head arthroplasty was performed in patients with larger lesions.

Regarding the CT results at the 5-year follow-up, the average bone graft resorption rate was 4% ± 2.4. In our opinion, this low percentage of bone resorption is to the result of the type of allograft (fresh-frozen) and the treatment of defects affecting up to 35% of the humeral head using small segmental allografts. In fact, allograft resorption may be caused by a lack of vascularization as well as immune responses, which can reduce the osteogenic capability and result in the production of antibodies against bone proteins [17]. Additionally, the graft source and method of graft preservation may have an impact: freezing and thawing allografts reduce the viability of passenger cells within the graft [18].

Some limitations of this study should be reported.

The first limitation was the lack of a control group.

The second limitation was the limited number of patients included in the study. Moreover, patients.

were prospectively reported but retrospectively evaluated. According to Gerber et al. [4] and Diklic et al. [14]., there are few studies in the literature discussing the treatment of chronic locked posterior shoulder dislocations. Moreover, only patients with limited bone defects were included in this study, thus further limiting the number of considered patients.

The rarity of the pathology should, however, be considered to partially explain the restricted number of patients included in the study.

Further studies of larger populations should be conducted to confirm these results.

## Conclusion

Segmental humeral head reconstruction with humeral head fresh-frozen osteochondral allografts provides good to excellent clinical results with low-grade OA and low allograft resorption in patients with locked posterior shoulder dislocation after a medium of 66 months.

## Data Availability

The datasets used and analysed during the current study are available from the corresponding author on reasonable request.

## Abbreviations

OA: Osteoarthritis
CA: cartilage angle
DA: defect angle
MRI: magnetic resonance imaging
CT: Computed tomography
WOSI: Western Ontario Shoulder Instability index
CS: the Constant-Murley score
ASES: American Shoulder and Elbow Surgeons Shoulder Score
ROCF: radius of curvature in the frontal plane
ROCS: radius of curvature in the sagittal plane
